# The Association Between Psychological Disorders and Physical Activity Among Saudi Women at Princess Nourah Bint Abdulrahman University

**DOI:** 10.7759/cureus.19413

**Published:** 2021-11-09

**Authors:** Sultana H Alsulaiman, Raghib Abu-Saris

**Affiliations:** 1 Health Sciences Research Center (HSRC), Princess Noura Bint Abdulrahman University, Riyadh, SAU; 2 Epidemiology and Biostatistics, King Saud Bin Abdulaziz University for Health Sciences, Riyadh, SAU

**Keywords:** princess nourah bint abdulrahman university, pnu, saudi women, sedentary behaviour, physical activity, stress, anxiety, depression

## Abstract

Introduction

Psychological disorders are one of the leading causes of disability globally. Evidence suggests the positive role of physical activity on physical and mental health in various countries. However, a limited number of studies have been done in Saudi Arabia to investigate the association between physical activity and psychological health, especially among women.

Objectives

The objective of the study was to identify the prevalence of depression, stress, anxiety, and physical activity as well as examine the association between depression, anxiety, stress, and social support in relation to physical activity among Saudi women at Princess Nourah Bint Abdulrahman University (PNU).

Methods

A total of 712 Saudi students and staff from PNU, with a mean (SD) age of 20.59(5.30), voluntarily completed an electronic-based questionnaire, which included three validated scales in the Arabic language: The Depression, Anxiety and Stress Scale - 21 Items (DASS-21), Global Physical Activity Questionnaire (GPAQ), and Medical Outcomes Study (MOS) social support.

Results

The results of this cross-sectional study showed that the prevalence of depression, anxiety, and stress were 24%, 31%, and 22.1%, respectively. Levels of physical activity were not associated with psychological disorders while sedentary time was strongly correlated with depression, anxiety, and stress (p<0.001). Emotional support, affectionate support, and positive social interaction were negatively correlated with depression (p<0.05).

Conclusion

The findings indicate that stress, depression, and anxiety were relatively common among Saudi females, especially among young ages, and that social support was a significant protective factor of depression. Community-based psychological health prevention programs and social support groups are needed to address these issues. The second major finding was that 51% of the participants were insufficiently active and 61% were having a sedentary lifestyle. Depression, anxiety, and stress scores are strongly correlated with sedentary time, which highlights the need for establishing a national policy that encourages active living and discourages sedentary behavior.

## Introduction

Psychological disorders are one of the leading causes of disability globally. According to WHO, mental and substance use disorders account for about 23% of all years lost due to disability [1]. Anxiety disorders are the most common mental disorders. WHO stated that one in 13 individuals suffers from anxiety worldwide [2]. Moreover, epidemiological studies have shown that 30 % of the population is affected by anxiety over their lifetime [3]. Depression is defined as a “mental disorder characterized by loss of interest, lack of activity, tiredness and fatigue, a change in appetite; sleeping more or less, feeling of worthlessness, and self-blame” [4]. It is a common mental illness that negatively affects how a person feels, thinks, and acts [5].

More than 264 million people worldwide are diagnosed with depression, and it is considered a major contributor to the global burden of disease [6-7]. Many studies were conducted on the prevalence of depression in Saudi Arabia but differ by population, gender, age group, and geographical location. According to these studies, the estimated prevalence was found to be between 17-46% [8], the prevalence was higher in women.

In 2010, Al Ibrahim et al. conducted a systematic review on depression, the prevalence found to be higher among women than men [9]. Literature has shown the positive role of physical activity on physical and mental health. There is strong evidence that shows daily physical activity can reduce the risk of depression and dementia for adults by 20-30% [10]. A systematic review conducted in 2011 found that physical activity and exercise have a beneficial effect on reducing symptoms of depression [11]. Moreover, a number of observational studies about the effect of physical activity on physiological health have been conducted locally. One study, including 870 university students, found that self-reported walking exercise was significantly associated with reduced symptoms of depression [12]. Another study has found that increased physical activity level was associated with better mental health [13]. Although the benefits of physical activity in general health and wellbeing are known, the prevalence of physical inactivity worldwide among adults is more than 80% [14]. In addition, the majority of the Saudi population is insufficiently physically active.

The prevalence of physical inactivity in Saudi Arabia ranged from 26% to 85% in males and it’s significantly higher among females (43% to 91%). Cultural barriers, lack of social support, the absence of female school physical activity programs, and the lack of sports facilities retrieved as barriers to physical activity among females. In contrast, the most motivators to exercise were health, losing weight, and socializing [15]. There is a need to increase the knowledge about physical activity and its benefits to psychological health. One study conducted among 800 Saudi women showed that 77.2% had poor practice patterns of physical activity, i.e., do not perform the recommended level of regular physical activity, and 64% of the participants had poor knowledge. Among them, 12.5% reported that physical activity does not prevent or decrease depression [16].

Although there are many studies in several countries conducted about the relationship between physical activity and mental health, to our knowledge, there are limited studies conducted locally that examine this association, especially among females. Therefore, the aim of this study is to assess the relationship between physical activity and psychological disorders among Saudi women at Princess Nourah Bint Abdulrahman University (PNU) in Riyadh, Saudi Arabia. The objective of the study was to identify the prevalence of depression, stress, anxiety, and physical activity, as well as examine the association between depression, anxiety, stress, and social support in relation to physical activity among Saudi women at Princess Nourah Bint Abdulrahman University. To be specific, the main objective of the study was to identify the prevalence of depression, stress, anxiety, and physical activity, as well as examine the association between depression, anxiety, stress, and social support, in relation to physical activity among Saudi women at Princess Nourah Bint Abdulrahman University. In particular, we formulated the following research questions:

1. What is the prevalence of physical activity, depression, anxiety, and stress among PNU students and staff?

2. In addition to physical activity and social support, what are potential risk factors for depression, anxiety, and stress among PNU students and staff?

3. Is there an association between physical activity and social support?

## Materials and methods

Study design 

This was an observational analytical study based on a cross-sectional design. The study was conducted at Princess Nourah Bint Abdulrahman University, the world’s largest women's university located in Riyadh, Saudi Arabia. There are 16 colleges offering different specializations. PNU has the capacity to enroll around 50,000 students and over 2000 faculty members [17].

Sampling and sample size

To estimate a prevalence using a 95% CI with a margin of error of 5%, the minimum sample size required was 385. Yet, after obtaining ethical approval from the PNU Research Ethical Committee, the final study sample comprised 712 Saudi female students and staff aged 18-55 years old.

The study used a convenience sampling technique, participants were asked to voluntarily participate in the study. An electronic self-administered questionnaire was sent through PNU's media email to all students and staff. Data were collected from 30 August 2020 to 30 September 2020.

Data collection tool

The first section of the questionnaire contains questions about demographic characteristics such as age, marital status, job status, and faculty or program. The second section included questions from the following validated scales in the Arabic language:

Depression, Anxiety and Stress Scale (DASS)

This is a self-report tool designed to measure the three related negative emotional states of depression, anxiety, and stress. It contains 21 items on a four-point Likert scale (0=Did not apply to me at all, 1=applied to me to some degree or some of the time, 2=applied to me to a considerable degree or a good part of the time, 3=applied to me very much or most of the time) [18]. Furthermore, the internal consistency, measured by Cronbach alpha, for depression, anxiety, and stress, is .879, .763, and .865, respectively. 

Global Physical Activity Questionnaire (GPAQ)

One of the most well-known self-reported tools for assessing health-related physical activity in different settings, including work, transportation, and leisure time, as well as time spent sitting (sedentary behavior) [19].

Medical Outcomes Study (MOS) Social Support Scale

This is a tool that measures perceptions and satisfaction with social support, which covers four domains (emotional/informational support, tangible support, positive social interaction, and affectionate support) [16]. It contains 19 items on a five-point Likert scale (1=Not at all, 2=A little of the time, 3=Some of the time, 4=Most of the time, and 5=All of the time) [20]. In addition, the internal consistency, measured by Cronbach alpha, for the first three domains is .931, ..891, and .928, respectively.

The questionnaire has been designed and sent electronically through Research Electronic Data Capture (REDCap) software, browser-based software for designing clinical and translational research databases (Vanderbilt University, 2004).

Data management and analysis

Data management and analysis were performed using Statistical Packages for Social Sciences (SPSS) version 24 (IBM Corp., Armonk, NY). Categorical variables were presented as frequencies and percentages, whereas continuous variables were presented as means and standard deviation. The chi-squared test was carried out to assess the association between baseline characteristics and physical activity levels and psychological disorders. A generalized linear model was constructed to examine the correlation between depression, anxiety, and stress scores (dependent variables) and physical activity levels and social support scores (independent variables). Statistical significance was determined by p-values less than .05.

## Results

Demographic characteristics of the study sample

The survey was sent to all PNU users, including students and faculty members. The total number of respondents was 904 but due to missing and duplicate data, the total sample size has eventually decreased to 712. The mean (SD) age of the respondents was 20.59 (5.30) of whom the majority were in the ≤ 20 age group (76.3%). Most of the participants were students and single (91%) (89%), respectively. Two-thirds of the students were in the first or second academic year (67.6%). Among the staff, 73% had bachelor's degrees while 18% had higher education. More details of the baseline characteristics are shown in Table 1.

**Table 1:** Demographic characteristics of study participants (N=712)

	N (%)	Mean (SD)	Median (IQR)	Min-Max
Age		20.59(5.30)	19(2)	17-50
≤ 20	495(76.3)			
21-30	110(16.9)			
31-40	33(5.1)			
≥ 41	11(1.7)			
Job status				
Students	646(90.7)			
Staff	66(9.3)			
Marital status				
Single	635(89.4)			
Married	58(8.2)			
Divorced	12(1.7)			
Separated	4(0.6)			
Widowed	1(0.1)			
Students’ family status				
Married and live together	513(80.3)			
Divorced	38(5.9)			
Separated	36(5.6)			
Other	52(8.1)			
Staff education level				
Secondary	6(9.1)			
Bachelor	48(72.7)			
Higher education	12(18.2)			
Students level				
Preparatory to first year	431(67.6)			
Second to third year	112(17.6)			
Forth to fifth year	62(9.7)			
Sixth to 7^th^ year	33(5.1)			
Students’ colleges				
Colleges of humanities	201 (32.1)			
Collages of sciences	197 (31.5)			
Collages of health sciences	130 (20.8)			
Colleges of community	98 (15.7)			
Smoking status				
Smokers	19(2.7)			
Non-smokers	688(97.3)			
Family monthly income				
Less than 10000 SAR	173 (24.6)			
10000-15000 SAR	127 (18)			
15001-20000 SAR	88 (12.5)			
20001-25000 SAR	44 (6.3)			
25001-30000	14 (2)			
More than 30000	46 (6.5)			
Don't know or don't want to answer	212 (30.1)			

Psychological characteristics of the study sample

Mild to moderate levels of depression, anxiety, and stress were found in 23%, 28%, and 22% of the study sample. The average (SD) for total social support score was 61.59 (20.26), that for emotional/informational support was 24.73 (9.61, tangible support was 14.25 (4.90), affectionate support was 9.68 (4.09), and positive social interaction was 9.97 (3.85); Table 2. Table 5 shows that students had higher levels of anxiety as compared to staff (P<0.01). Moreover, a significant association was detected between stress and anxiety with age and student’s academic level, where younger age groups tended to have mild to moderate levels of stress and anxiety symptoms compared to older age groups (P=0.005) (P=0.007), respectively. In addition, students in the preparatory to the first years were more likely to develop stress and anxiety (P=0.030) ( P=0.007), respectively. Single participants were more prone to stress (P=0.034). A significant association was also found between smoking status and anxiety and depression, P-values = 0.037 and 0.003, respectively. The total social support score, on the other hand, was not associated with demographic characteristics.

**Table 2:** Psychological characteristics of study participants

	N (%)	Mean (SD)	Median (IQR)	Min-Max
Depression (n=709)		6.07(5.20)	5(7)	0-21
No depression	543(76.6)			
Mild	77(10.9)			
Moderate	85(12)			
Severe	4(0.6)			
Anxiety (n=709)		5.02(4.19)	4(5)	0-18
No anxiety	488(68.8)			
Mild	111(15.7)			
Moderate	86(12.1)			
Severe	24(3.4)			
Stress (n=687)		6.88(5.13)	6(7)	0-21
No stress	528(77.9)			
Mild	126(18.6)			
Moderate	24(3.5)			
Social support score		61.59(20.26)	62(32)	19-95
Emotional/informational support		24.73(9.61)	24(17)	8-40
Tangible support		14.25(4.90)	15(9)	4-20
Affectionate support		9.69(4.09)	10(8)	1-15
Positive social interaction		9.96(3.86)	10(7)	3-15

Physical activity and sedentary behavior

Table 3 represents physical activity levels among study participants. According to IPAQ, the mean (SD) physical activity metabolic equivalent minutes (MET)-minutes/week was 2728.65 (3749.97). Half of the study respondents were classified as low physically active (51.2%), 21.7% were classified as moderately physically active, while 27.2% were at a high level of physical activity. Regarding setting-specific physical activity, occupational physical activity has the highest mean score (64.32±107.36 mins/day), followed by recreational physical activity (47.92±51.62 mins/day). The mean (SD) setting time per day was 482.88 (321.77) (Table 4). More than half of the respondents (61%) reported being sedentary (setting for ≥ 6 hours per day) (Table 3).

**Table 3:** Levels of total physical activity among study participants MET: metabolic equivalent minutes

	N (%)	Mean(SD)	Median (IQR)
Total PA MET minutes/ week		2728.65(3749.97)	1560(2620)
Physical activity levels ^a^			
High	173(27.2)		
Moderate	138(21.7)		
Low	326(51.2)		
Total sitting time (Hours/Day)		8.05(5.36)	6(6)
≥ 6 hours	363(61)		
<6 hours	230(39)		
^a ^Low: <600MET min/week, moderate: 600–2999 MET min/week, high: ≥1500 MET min/week vigorous PA or ≥3000 MET min/week moderate/vigorous PA			

**Table 4:** Average total setting-specific physical activity in minutes per day

Setting-specific physical activity	N	Mean ± SD	Median (IQR)	95% CI of Mean
Work	230	64.32±107.36	34.28(51.43)	50.37-78.27
Transportation	186	32.59±46	17.14(33.57)	25.93-39.24
Recreation	301	47.92±51.62	34.28(45.71)	42.07-53.78
Sedentary	593	482.88±321.77	360(360)	456.93-508.83

Levels of physical activity were significantly associated with age, high to moderate levels of physical activity were more detected among the ≤ 20 age group compared to older age groups (P=0.04). Furthermore, staff with a bachelor's degree had higher levels of physical activity compared to those with secondary and higher education (P=0.04) (Table 5). There was no significant difference between baseline characteristics in regard to sedentary behaviors.

**Table 5:** Association between psychological disorders and baseline characteristics

	Normal N (%)	Mild N (%)	Moderate N (%)	Severe N (%)	P-value
Stress					
Job Status	N=528	N=126	N=24	-	0.142
Students	478(90.5)	119(94.4)	20(83.3)		
Staff	50(9.4)	7(5.6)	4(16.7)		
Age group	N=482	N=116	N=21	-	0.005
≤ 20	373(77.4)	86(74.1)	12(57.1)		
21-30	78(16.2)	25(21.6)	4(19)		
31-40	23(4.8)	4(3.4)	5(23.8)		
≥ 41	8(1.7)	1(0.9)	0(0)		
Marital status	N=526	N=126	N=25	-	0.034
Single	472(89.7)	115(91.3)	20(80)		
Married	44(8.4)	7(5.6)	3(12)		
Divorced	9(1.7)	1(0.8)	1(4)		
Separated	0(0)	3(2.4)	0(0)		
widowed	1(0.2)	0(0)	1(4)		
Student level	N=474	N=118	N=20	-	0.030
Preparatory to first year	328(69.2)	75(63.6)	9(45)		
Second to third year	77(16.2)	26(22)	5(25)		
Forth to fifth year	48(10.10	7(5.9)	5(25)		
Six to 7^th^ year	21(4.4)	10(8.5)	1(5)		
Smoking status	N=524	N=126	N=24	-	0.518
Smokers	12(2.3)	5(4)	1(4.2)		
Non-smokers	512(97.7)	121(96)	23(95.8)		
Anxiety					
Job status	N=488	N=111	N=86	N=24	0.003
Students	438(89.8)	103(92.8)	81(94.2)	21(87.5)	
Staff	50(10.2)	8(7.2)	5(5.8)	3(12.5)	
Age group	N=444	N=99	N=84	N=19	0.007
≤ 20	348(78.4)	70(70.7)	65(77.4)	10(52.6)	
21-30	63(14.2)	27(27.3)	14(16.7)	5(26.3)	
31-40	23(5.2)	2(2)	5(6)	3(15.8)	
≥ 41	10(2.3)	0(0)	0	1(5.3)	
Marital status	N=486	N=111	N=86	N=24	0.276
Single	431(88.7)	100(90.1)	80(93)	21(87.5)	
Married	42(8.6)	10(10)	5(5.8)	1(4.2)	
Divorced	11(2.3)	0(0)	0(0)	1(4.2)	
Separated	1(0.2)	1(0.9)	1(1.2)	1(4.2)	
Widowed	1(0.2)	0(0)	0(0)	0(0)	
Student level	N=431	N=103	N=80	N=21	0.007
Preparatory to first year	307(71.2)	61(59.2)	51(63.8)	12(57.1)	
Second to third year	69(16)	20(19.4)	18(22.5)	3(14.3)	
Forth to fifth year	38(8.8)	17(16.5)	4(5)	2(9.5)	
Six to 7^th^ year	17(3.9)	5(4.9)	7(8.8)	4(19)	
Smoking status	N=484	N=111	N=85	N=24	0.037
Smokers	9(1.9)	3(2.7)	6(7.1)	0(0)	
Non-smokers	475(98.1)	108(97.3)	79(92.9)	24(100)	
Depression					
Job status	N=543	N=77	N=85	N=4	0.606
Students	489(90.1)	72(93.5)	78(91.8)	4(100)	
Staff	54(9.9))	5(6.5)	7(8.2)	0(0)	
Age group	N=492	N=70	N=81	N=3	0.749
≤ 20	382(77.6)	54(77.1)	55(67.9)	2(66.7)	
21-30	77(15.7)	12(17.1)	19(23.5)	1(33.3)	
31-40	24(4.9)	4(5.7)	5(6.2)	0(0)	
≥ 41	9(1.8)	0(0)	2(2.5)	0(0)	
Marital status	N=541	N=77	N=85	N=4	0.470
Single	480(88.7)	73(94.8)	75(88.2)	4(100)	
Married	49(9.1)	2(2.6)	7(8.2)	0(0)	
Divorced	10(1.8)	1(1.3)	1(1.2)	0(0)	
Separated	1(0.2)	1(1.3)	2(2.4)	0(0)	
widowed	1(0.2)	0(0)	0(0)	0(0)	
Student level	N=482	N=72	N=77	N=4	0.260
Preparatory to first year	336(69.7)	47(65.3)	46(59.7)	2(50)	
Second to third year	79(16.4)	16(22.2)	14(18.2)	1(25)	
Forth to fifth year	45(9.3)	4(5.6)	12(15.6)	0(0)	
Six to 7^th^ year	22(4.6)	5(6.9)	5(6.5)	1(25)	
Smoking status	N=538	N=77	N=85	N=4	0.003
Smokers	11(2)	5(6.5)	1(1.2)	1(25)	
Non-smokers	527(98)	72(93.5))	84(98.8)	3(75)	

Physical activity, sedentary behavior, and psychological disorders

Tables 6-7 show the linear correlation between psychological disorders and physical activity levels. A number of possible confounders including age, job status, marital status, students’ academic level, sedentary time, and social support scores were included in the model. In the unadjusted analysis, higher levels of physical activity were significantly correlated with lower depression symptoms score (P=0.029); however, this difference was not statistically significant after controlling for sedentary time (P=0.447). No significant correlation was found between stress and anxiety scores and physical activity levels either. Sedentary time, on the other hand, was strongly associated with stress, anxiety, and depression (P<0.001). Higher levels of affectionate support were associated with lower stress and depression scores (P=0.057) (P=0.009), respectively, while the emotional/informational and positive social interaction kinds of support were significantly associated with lower levels of depression (P=0.036) (P=0.017). All kinds of social support were not significantly associated with levels of physical activity.

**Table 6:** Factors associated with physical activity level among study participants

	Physical activity levels N (%)			P-value
	High	Moderate	Low	
Age group				0.046
≤20	223(75.6)	92(73)	132(81.5)	
21-30	43(14.6)	28(22.2)	25(15.4)	
31-40	23(7.8)	4(3.2)	3(1.9)	
≥41	6(2)	2(1.6)	2(1.2)	
Staff education level				0.042
Secondary	0(0)	0(0)	6(15)	
Bachelor	9(90)	11(100)	23(57.5)	
Higher education	1(10)	0(0)	11(27.5)	

**Table 7:** Association between psychological disorders, levels of physical activity, and social support using the generalized linear model analysis ^a^ Minutes spent sitting per day

		B (Coefficient)		Std. Error		Wald Chi-Square	95% Wald CI			P-Value	
							Lower Bound; Upper Bound				
Stress											
Physical activity level											
High		.137		.5080	.073			-.859	1.133	.787	
Moderate		-.878		.5496	2.553			-1.956	.199	.110	
Low		0^a^		.	.			.	.	.	
Total social support score		.383		.2412	2.518			-.090	.856	.113	
Emotional/informational support		-.375		.2496	2.251			-.864	.115	.134	
Tangible support		-.411		.2468	2.768			-.894	.073	.096	
Affectionate support		-.513		.2691	3.627			-1.040	.015	.057	
Positive social interaction		-.675		.3113	4.701			-1.285	-.065	.030	
Sedentary time^a^		.171		.0407	17.784			.092	.251	< .001>	
Anxiety											
Physical activity level											
High			.347	.4269	.662		-.489		1.184		.416
Moderate			-.195	.4635	.177		-1.104		.713		.674
Low			0^a^	.	.		.		.		.
Total social support score	.030			.2033	.021		-.369		.428		.884
Emotional/informational support	-.033			.2105	.024		-.445		.380		.877
Tangible support	-.134			.2090	.412		-.544		.276		.521
Affectionate support	.041			.2257	.032		-.402		.483		.857
Positive social interaction	-.232			.2627	.777		-.747		.283		.378
Sedentary time^a^	.183			.0341	28.961		.117		.250	<0.001	
Depression											
Physical activity level											
High		-.183		.4643		.155	-1.093		.727	.694	
Moderate		-.626		.4935		1.608	-1.593		.342	.205	
Low		0a				.	.		.	.	
Total social support score		.395		.2196		3.237	-.035		.825	.072	
Emotional/informational support		-.479		.2281		4.406	-.926		-.032	.036	
Tangible support		-.335		.2256		2.202	-.777		.107	.138	
Affectionate support		-.636		.2430		6.847	-1.112		-.160	.009	
Positive social interaction		-.675		.2827		5.706	-1.229		-.121	.017	
Sedentary time^a^		.247		.0369		44.779	.175		.319	< .001>	

## Discussion

The purpose of this study was to enrich the epidemiological data on the prevalence and determinants of psychological disorders and physical activity among Saudi women at Princess Nourah Bint Abdulrahman University. With respect to the first research question, it was found that the prevalence of depression symptoms, anxiety symptoms, and stress was 24%, 31%, and 22.1%, respectively. Given the variations in the measuring scales and characteristics of the sample, our findings were similar to those obtained in a study conducted in Saudi Arabia [21]. Stress and anxiety were associated with younger age groups, this finding is consistent with that of Mirzaei et al. (2019) and Bandelow (2015), which indicated that younger age groups were more stressed compared to older age groups [22], and anxiety disorder decreases with older age [3]. This could be also due to the fact that most of our sample were within younger ages. Anxiety was more observed among students compared to staff, which may be explained by the fact that most of the students were in their first year, which is probably a high-risk time for anxiety. Our study confirms that smoking is associated with depression and anxiety, this finding was also reported by Mykletun et al. (2008) [23]. Regarding social support, previous studies have established the relationship between social support and mental health, suggesting that the higher level of social support, the lower level of psychological problems [24-25]. The present study supports this evidence, which found that several types of social support, including emotional/informational support, affectionate support, and positive social interaction, were negatively correlated with the depression score.

Low physical activity level was reported by 51% of the sample. In addition, older age groups had significantly lower levels of PA. These results reflect the previous literature that showed a high prevalence of physical inactivity among Saudi females (43%-91%) [15] and that inactivity increases with age [26].

The present study has revealed that physical activity levels were not associated with any psychological disorders, this finding is contrary to previous studies that have suggested that there is a positive role of physical activity on mental health [10-12]. This could be attributed to our study population comprising educated females only. In any case, the most obvious finding to emerge from the analysis is that majority of participants were sedentary; the mean (SD) of sitting time was 8.05 (5.36) hours per day. In addition, more than half of the participants reported sitting for ≥ 6 hours per day. These results were in agreement with those obtained in recent studies, which reported a high frequency of sedentary time among Saudi college female students [27] and among Saudi women working in office-based jobs [28].

Although physical activity was not found to be a predictor of psychological disorders, sedentary behavior was strongly correlated with higher levels of stress, anxiety, and depression symptoms. This study supports evidence from recent studies, which showed that as sitting time increases, levels of stress, anxiety, and depression increase among college students [29-30].

Finally, a number of limitations need to be noted. First, the current study was observational in nature, hence, the dose-response relationship cannot be proven. Second, though the surveys used were valid and reliable, the responses relating to physical activity, psychological disorders, and social support were subjective and were therefore susceptible to social desirability bias. Third, the lack of random selection of the sample and the fact that it was restricted to one university in Riyadh adds further caution regarding the generalizability of these findings.

## Conclusions

This study set out to determine the prevalence of physical activity and psychological disorders among educated Saudi women and to examine the role of physical activity on the symptoms of psychological disorders. The findings indicate that stress, depression, and anxiety were relatively common among educated Saudi females, especially among young ages, and that social support was a significant protective factor of depression. Community-based psychological health prevention programs and social support groups are needed to address these issues. The second major finding was that 51% of the participants were insufficiently active. Moreover, prolonged sitting time was significantly correlated with depression, anxiety, and stress symptoms. The current data highlight the importance of physical activity promotion programs and establishing a national policy that encourages active living and discourages sedentary behavior. A further retrospective cohort study should be a better design to examine the effect of physical activity and sedentary lifestyle on psychological disorders among women in Saudi Arabia.
